# Gas Sensor Based on Photonic Crystal Fibres in the 2*ν*_3_ and *ν*_2_ + 2*ν*_3_ Vibrational Bands of Methane

**DOI:** 10.3390/s90806261

**Published:** 2009-08-10

**Authors:** Ana M. Cubillas, Jose M. Lazaro, Olga M. Conde, Marco N. Petrovich, Jose M. Lopez-Higuera

**Affiliations:** 1 Photonics Engineering Group, University of Cantabria, Avda. de los Castros S/N, 39005 Santander, Spain; E-Mails: josemiguel.lazaro@unican.es (J.M.L.); olga.conde@unican.es (O.M.C.); miguel.lopezhiguera@unican.es (J.M.L.-H); 2 Optoelectronics Research Centre, University of Southampton, Southampton, SO17 1BJ, UK; E-Mail: mnp@orc.soton.ac.uk (M.N.P.)

**Keywords:** gas detectors, methane, fibre optic sensors, photonic crystal fibres, hollow-core photonic bandgap fibres, absorption spectroscopy

## Abstract

In this work, methane detection is performed on the 2*ν*_3_ and *ν*_2_ + 2*ν*_3_ absorption bands in the Near-Infrared (NIR) wavelength region using an all-fibre optical sensor. Hollow-core photonic bandgap fibres (HC-PBFs) are employed as gas cells due to their compactness, good integrability in optical systems and feasibility of long interaction lengths with gases. Sensing in the 2*ν*_3_ band of methane is demonstrated to achieve a detection limit one order of magnitude better than that of the *ν*_2_ + 2*ν*_3_ band. Finally, the filling time of a HC-PBF is demonstrated to be dependent on the fibre length and geometry.

## 1. Introduction

Methane (CH_4_) is a very common gas, found in many industrial applications where it carries a significant explosion hazard, and in the environment where it can give rise to strong greenhouse effect contributing to global warming. It is therefore of great interest to develop methane sensors to accurately monitor its concentration [1]. The most common methane sensors are pellistor or catalytic detectors, which detect methane by the ignition of the gas on a heated filament. These sensors are cheap and easy to use, but at the same time, they are very sensitive to surface poisoning, prone to calibration drifts and lack selectivity [1, 2].

Optical absorption spectroscopy, on the other hand, is very well suited for methane sensing due to its high spectral resolution, high selectivity, immunity to poisoning and electromagnetic linebreak interferences [3]. Furthermore, fibre-based detection systems offer additional advantages over conventional systems such as small size, light weight and possibility of remote and distributed measurements [4]. Up to date, different conventional fibre designs have been investigated as gas cells but, in most cases, they failed to achieve sufficient detection sensitivity [4, 5].

Recently, Microstructured Optical Fibres and, in particular, Hollow-core Photonic Bandgap Fibres (HC-PBFs) have proved their potential for gas sensing applications [5–9]. When the hollow core of these fibres is filled with gas, long interaction lengths between light and the gas can be readily achieved, thus enabling high detection sensitivity. Furthermore, HC-PBFs can be coiled, achieving very compact devices, and can be integrated with conventional fibres [10, 11].

In the Near-Infrared (NIR) wavelength region, methane has two vibrational absorption bands: the *ν*_2_ + 2*ν*_3_ combination band, centered at 1.33 *μ*m, and the 2*ν*_3_ overtone band, at 1.66 *μ*m [12]. Previously, methane has been detected in these bands using conventional cells [1, 13] and HC-PBFs [7, 8]. In this work, the results of methane detection using HC-PBFs specifically designed for the two vibrational bands are compared. The feasibility as well as the benefits and drawbacks of methane sensing in either regions are discussed. Furthermore, the dynamics of the filling processes of HC-PBFs as a function of the structure and length of the fibres are investigated.

## 2. Fundamentals of Absorption Spectroscopy

The fundamental principle of absorption spectroscopy is the Beer-Lambert law [3]. This states that the absorbance of light through a gas, *α*(*ν*), at a frequency *ν* [cm*^−^*^1^], is related to the properties of the gas species through which the light is traveling by the following expression:

(1) $$\alpha (\nu )=-\text{ln}\left(\frac{I_{t}}{I_{o}}\right)=\sum_{i=1}^{N}S_{i}(T)\phi_{i}(\nu )\mathrm{pcL}$$

where the absorbance depends on the ratio between the light transmitted through the gas, *I_t_*, and the incident light, *I_o_*. Furthermore, *N* represents the number of transitions within a gas line manifold, *i* a specific transition, *p* [atm] the total pressure of the medium, *c* the mole fraction of the gas species (related to the gas concentration), *S_i_*(*T*) [cm*^−^*^2^atm*^−^*^1^] the linestrength for transition *i* at a temperature *T*, *ϕ_i_*(*ν*) [cm] the normalized lineshape function and, finally, *L* [cm] represents the path length, i.e., the distance light travels through the gas. The absorbance is usually expressed in absorbance units (AU). Additionally, the fraction of the transmitted light and the incident light is referred as transmittance, *T* (*ν*).

The lineshape function, *ϕ*(*ν*), is represented by a Gaussian profile when the effects of line broadening are due to thermal motion (Doppler broadening). Alternatively, collision effects, due to pressure, give rise to a Lorentzian lineshape function (collisional broadening). When both effects are significant, the resultant lineshape function is typically addressed using a Voigt profile [14].

## 3. Methane Absorption Bands in the NIR

The majority of gases of interest, like methane, present their strongest molecular absorption lines in the Mid-Infrared (MIR) wavelength region (specifically, in the 3 to 5 *μ*m range). Unfortunately, MIR light sources in this range are generally inefficient and MIR detectors are also expensive and require liquid nitrogen cooling, consequently cumbersome to operate. On the other hand, in the NIR region, a great variety of high-performance telecommunication sources and detectors are available at lower cost. Furthermore, operation in the NIR region opens up the possibility for remote sensing applications thanks to the compatibility with standard telecommunication fibres. For these reasons, NIR region has been the preferred option in spectroscopic systems, despite exploiting weaker absorption lines and sacrificing sensitivity [3].

**Figure 1.**
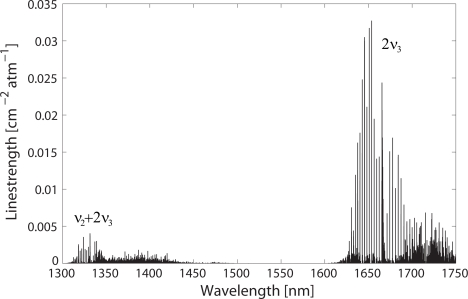
Linestrengths of the *ν*_2_ + 2*ν*_3_ and 2*ν*_3_ vibrational bands of methane in the NIR. Reproduced from [12].

In the NIR, methane has two main wavelength regions: *ν*_2_ + 2*ν*_3_ combination band, at 1.33 *μ*m, and 2*ν*_3_ overtone band, at 1.66 *μ*m. The absorption lines composing these bands, obtained from the HITRAN database [12], are plotted in Figure 1.

As can be seen from the plot, the *ν*_2_ +2*ν*_3_ band of methane comprises very weak absorption lines. Additionally, water absorption is also present in this region [12] and a suitable absorption line of methane free from water interference needs to be selected, which significantly adds to the challenge. On the other hand, the 1.33 *μ*m wavelength region benefits from the high-performance and cost-effective light sources and detectors which are available for the second telecommunication window. The long interaction lengths possible by using HC-PBFs could therefore help alleviate the issue of the low absorption linestrength and allow for methane sensors with a good trade-off between cost and sensitivity.

Conversely, the strongest absorption band of methane in the NIR is the 2*ν*_3_ band, located at 1.66 *μ*m (see Figure 1). Here, absorption lines are approximately one order of magnitude stronger as compared with the *ν*_2_ + 2*ν*_3_ band. For this reason, most of the NIR methane gas sensors based on conventional, free-path cells generally target this band [1].

Use of HC-PBFs custom designed for operation at 1.66 *μ*m may open up the possibility of methane sensors with very high sensitivity, although it has to be noted that telecom laser sources and detectors at these longer wavelengths are not as readily available and cheap as in the 1.33 *μ*m region. However, it is expected that the need for wider operational bandwidth of telecom networks will shortly drive the development of new and better sources and detectors, which could benefit methane detection in the 2*ν*_3_ band [15].

## 4. Materials and Methods

Two different HC-PBFs, designed specifically to operate at the two principal NIR vibrational bands of methane, were used in this work. In the following, the fibres are labeled as HC1300 and HC1600, according to their transmission bands. The specifications of both HC-PBFs are detailed in Table ??.

**Figure 2.**
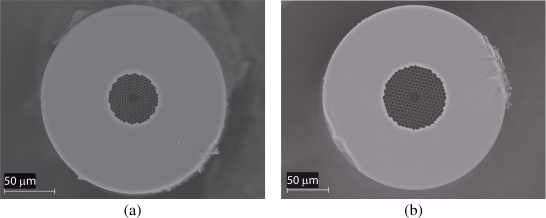
SEM micrographs of the cross-section of the (a) HC1300 fibre and (b) HC1600 fibre.

**Figure 3.**
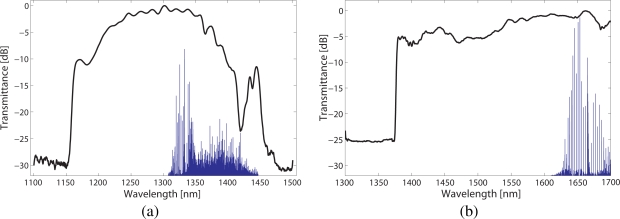
Normalized spectral transmission (solid black) and methane linestrengths (solid blue) of (a) a 5.6 m long HC1300 and methane lines at *ν*_2_ + 2*ν*_3_ band, and (b) a 4.9 m long HC1600 and methane lines at 2*ν*_3_ band.

Both fibres were manufactured by the Optoelectronics Research Centre, University of Southampton, using a two-step stack-and-draw process [11]. The microstructure of the both fibres is composed of a triangular lattice of air holes, in which the core is formed by removing seven capillaries from the centre. High-resolution scanning electron microscope (SEM) images of both fibre cross-sections are illustrated in Figure 2. The structural parameters of the fibres were determined from these pictures. Figure 3 (a),(b) shows the normalized spectral transmission of the HC1300 and HC1600 fibres, from which it is evident the presence of bandgaps at the respective target wavelengths. Also shown in Figure 3 (a) and (b) are the normalized linestrengths of methane in the *ν*_2_ + 2*ν*_3_ and 2*ν*_3_ bands [12], which provides a clear demonstration that the two fibres are suited for the detection of methane in the respective bands.

**Figure 4.**
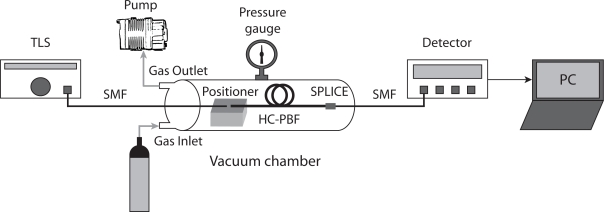
Experimental setup for methane detection experiments using HC-PBF as gas cells.

Figure 4 provides a scheme of the scanned-wavelength direct absorption spectroscopy system used for methane detection. Light from a tunable laser source (TLS) was launched into a Single Mode Fibre (SMF). The SMF was butt-coupled to the HC-PBF using a 3-axis positioner. A gap was left between the ends of the fibres to allow the gas access into the core of the HC-PBF. The other end of the HC-PBF was spliced to a SMF pigtail using a similar procedure as described in [16]. The splice attenuation was measured to be about 1 dB. The light transmitted light through the HC-PBF was measured using a Ge detector. The open end of the HC-PBF was placed inside a sealed vacuum chamber, as illustrated in Figure 4. Finally, a pump was used to evacuate the fibre prior to filling with calibrated concentrations of the target gas.

Two slightly different systems were set up for the detection at the different vibrational bands of methane. For detection at the *ν*_2_ + 2*ν*_3_ vibrational band, we used a 5.6 m long piece of HC1300 fibre was employed as the gas cell, an Agilent 8167B tunable laser (wavelength range 1,255–1,365 nm, linewidth 100 KHz), and an Agilent 8163A power meter as the detector. On the other hand, for the 2*ν*_3_ band, the gas cell (HC1600) was 5.1 m long, a Santec TSL-210V tunable laser was used as the source (wavelength range 1,580–1,680 nm, linewidth 1 MHz) and the same optical power meter, Agilent 8163A, was utilized to measure the transmitted power through the fibre cell.

## 5. Experimental Results

### 5.1. Methane Absorption Spectrum Measurements

The first experiment was to measure methane absorption spectrum using the two HC-PBF cells and the setup described in the previous section. For this purpose, the vacuum chamber was first evacuated and then filled with a calibrated mixture of 18,750 ppmv (parts per million volume) methane in air at a relative pressure of 1 bar. After allowing sufficient time for the gas to completely diffuse into the fibre, a transmission spectrum was recorded. The measured spectrum was then normalized to a spectrum collected with empty cells, yielding the absorption due to methane.

**Figure 5.**
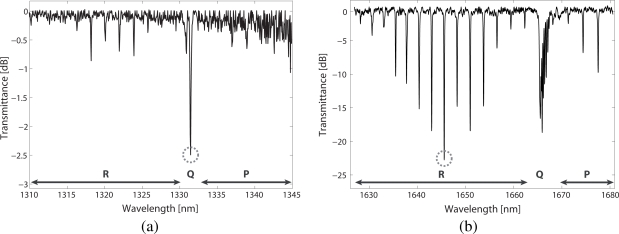
Transmission spectrum of methane at (a) the *ν*_2_ + 2*ν*_3_ band, measured with the HC1300 fibre, and (b) at 2*ν*_3_ band, measured with the HC1600 fibre. R, P and Q branches are labeled. The selected features for methane detection are circled.

Figure 5 (a) shows the *ν*_2_ + 2*ν*_3_ methane absorption band. The methane spectrum in this region is very complex and composed of many closely spaced and generally very weak lines. However, the R, Q and P branches can be clearly observed in the spectrum (see labels). The Q-branch, centered at 1331.55 nm (circled in the figure), is significantly stronger than the other peaks in the spectrum. This feature was therefore an obvious choice for our concentration measurements. Nevertheless, its major drawback is that it is in fact a very broad manifold, composed of more than 80 energy transitions [12]; its fine structure was not resolved in our measurements.

The methane absorption spectrum at the 2*ν*_3_ band is shown in Figure 5 (b). As noticed before, methane absorption lines are much stronger in this case as compared with the *ν*_2_ + 2*ν*_3_ band (notice the different vertical scales). R, Q and P branches are also labeled in the figure. For methane sensing purposes, the R(6) manifold, at 1645 nm, was targeted (circled in the figure) since it affords the best Sinal-to-Noise Ratio (SNR) in the band. Besides, this feature is narrower than the Q-branch as it is composed of 6 transitions [12]. Finally, in both cases, the resulting spectra was found to agree with HITRAN database absorption data [12].

### 5.2. Detection Limit of the System

**Figure 6.**
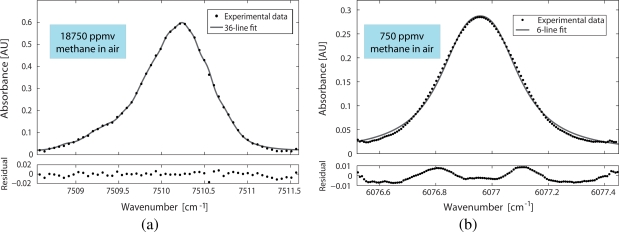
Analysis of the detection limit of methane: (Top) Experimental absorbance data and relative fit; (Bottom) Residuals of the fit. (a) 36-transition Lorentzian fit for the Q-branch at *ν*_2_ + 2*ν*_3_ band at room temperature, relative pressure of 1 bar and methane concentration of 18750 ppmv. (b) 6-transition Lorentzian fit for the R(6) manifold at 2*ν*_3_ band at room temperature, relative pressure of 1 bar and methane concentration of 750 ppmv.

The detection limit of the system for the *ν*_2_ + 2*ν*_3_ and 2*ν*_3_ bands was calculated by analyzing the absorbance of the selected lines. The absorbance was therefore computed from the normalised transmitted intensities through the HC-PBF cells (using Equation 1). These data points are plotted in the upper panels of Figure 6 as dots. For the Q-branch at *ν*_2_ + 2*ν*_3_ band, they were obtained using a 18,750 ppmv sample of methane at room temperature and a relative pressure of 1 bar; for the R(6) manifold at 2*ν*_3_ band, the gas sample used was a calibrated mixture of 750 ppmv methane in air.

Using the procedure described in [17], the detection limit for both absorption bands, *c_min_*, was calculated by extrapolation to SNR = 1 (see [7] and [8] for a more detailed explanation). In both cases, the limiting noise was mainly due to unwanted interference features from the HC-PBFs. For the Q-branch at *ν*_2_ + 2*ν*_3_ band, 36 energy transitions were needed to accurately fit the absorbance (Equation 1), and a detection limit of 98 ppmv was achieved [7]. In the case of R(6) line at 2*ν*_3_ band, only 6 transitions were considered, an a detection limit of 10 ppmv was estimated [8].

Taking into account the estimated detection limits of both systems, the minimum detectable absorbance (MDA) can be deduced using the following equation:

(2) $$\text{MDA}=\sum_{i=1}^{N}S_{i}(T)\phi_{i}(\nu ){\mathrm{pc}}_{\min}L$$

This implies that, with our arrangement, a MDA of 3.1 × 10^−3^ AU and a MDA of 8.0 × 10^−4^ AU can be measured at the Q-branch at *ν*_2_ + 2*ν*_3_ band and at the R(6) manifold at 2*ν*_3_ band, respectively. These results are slightly higher than typically reported values for direct absorption spectroscopy systems [18], due to the noise in our system. Finally, it can be concluded that the sensitivity results from the 2*ν*_3_-band HC-PBF significantly improve the ones obtained for the *ν*_2_ + 2*ν*_3_-band HC-PBF, due to the better absorption of the gas in the former band.

### 5.3. Study of the Dynamics of the Filling Process of HC-PBFs

Whilst HC-PBFs allow for very interaction lengths to be achieved, the use of long fibre lengths also carries the significant drawback of longer filling time. Indiffusion of target gas species in the holes of HC-PBFs is typically a slow process, in the order of minutes at best, which clearly affects the response time of the sensor. This point was confirmed experimentally by analysing the filling process of the different HC-PBFs used in the experiments.

**Figure 7.**
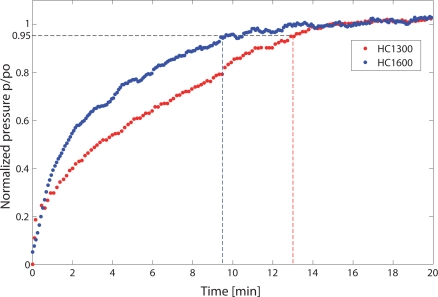
Measured filling process of the HC1300 (red dots) and HC1600 (blue dots) fibres as a function of time. The filling time is indicated with dashed lines.

The vacuum chamber was first evacuated and then filled with a 18,750 ppmv mixture of methane in air at ambient temperature and a relative pressure of 1 bar. The intensity transmitted through the HCPBF cells on the selected methane lines was continually monitored during the filling process. The normalised transmitted intensity was used to calculate the methane partial pressure inside the fibre through Equation 1; the latter was normalized to the equilibrium pressure value, *p*_0_, measured when the cell is completely filled with the target mixture. The resulting filling process for the different fibres is shown in Figure 7.

The filling time was defined as the time needed for the gas to reach a normalized pressure of 0.95. The filling time was measured to be approximately 13 and 9.5 minutes for the HC1300 and HC1600 fibre, respectively. As can be seen, the filling process takes longer in the HC1300 fibre than in the HC1600 fibre. We advanced the hypothesis that the longer filling time may be due to the fact that the HC1300 fibre has a smaller core diameter and a marginally longer length than the HC1600 fibre, and that the aspect ratio of the fibre (i.e. the ratio between the core and the length of the HC-PBF) greatly influences the filling process.

Indeed, the equations describing the transient flow of gases inside the core of HC-PBFs have been recently reported [19]. The nature of gas flow is characterized by the Knudsen number [20]. Under our experimental conditions, the Knudsen number is very small and the gas flow follows an hydrodynamic model. Therefore, the equation of the variation of the normalized pressure, *κ* = *p/p*_0_, as a function of the dimensionless time, *t_h_*, is [19]:

(3) $$\frac{\partial \kappa }{{\partial t}_{h}}=\frac{\partial }{\partial \xi }\left(\kappa \frac{\partial \kappa }{\partial \xi }\right)$$

where *ξ* is the normalized position, defined as *ξ* = *x/L*, and *L* is the total fibre length. Furthermore, the dimensionless time is related to the real time, *t*, through the expression:

(4) $$t=\frac{32\eta L^{2}}{d^{2}p_{0}}t_{h}$$

in which *η* is the dynamic viscosity of the gas and *d* is the core diameter of the fibre. Furthermore, the viscosity of methane at ambient temperature has been found to be 11.01 × 10^−6^ Pa*·*s [21].

**Figure 8.**
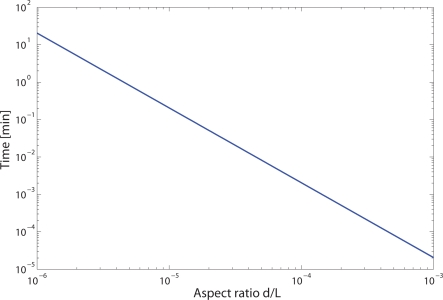
Filling time required for methane gas diffusion as a function of the aspect ratio *d/L* of the HC-PBF employed, in logarithm scales.

According to Equation 4, the gas flow inside HC-PBFs is highly dependent on the aspect ratio of the fibre, *d/L*. Therefore, the filling time was theoretically calculated for fibres with different aspect ratios. This was done by solving Equation 3 numerically, using two-open end boundary conditions, by integrating the solution across the spatial dimension, and then expressing the dimensionless time in real time units [19]. The result is shown in Figure 8. As expected, the filling time is highly dependent on the aspect ratio of the HC-PBF used. Therefore we concluded that, in order to reduce the filling times obtained in our measurement cells, different HC-PBF fibres with bigger core dimensions or shorter sample lengths would be required.

### 5.4. Summary of the Results

Table 2 summarizes the results obtained in this study for methane sensing at 1.33 *μ*m and 1.66 *μ*m regions and the main parameters of the two HC-PBFs used.

## 6. Conclusions

In this work, we have demonstrated methane sensing using Hollow-Core Photonic Bandgap Fibres as gas cells, targeting two different wavelength bands in the Near-Infrared Region, i.e. the *ν*_2_ + 2*ν*_3_ combination band, centered at 1.33 *μ*m, and the 2*ν*_3_ overtone band, at 1.66 *μ*m. The benefits and drawbacks of each wavelength region have been assessed. It has been demonstrated that, since 2*ν*_3_ band shows one order of magnitude stronger absorption lines than the *ν*_2_ + 2*ν*_3_ band, it also affords the highest detection sensitivity. More in detail, a low detection limit of 98 ppmv was achieved with the *ν*_2_ + 2*ν*_3_ band detection scheme, whereas a detection limit of 10 ppmv was achieved for the 2*ν*_3_ band. Although 1.33 *μ*m detection still holds some advantage due to the availability of laser sources, detection at 1.66 *μ*m allows for a significantly higher detection sensitivity. The response time of a methane gas sensor based on HC-PBFs has been studied by investigating the filling process. It has been demonstrated that the latter is strongly dependent on the aspect ratio of the fibre considered, when using vacuum or pressure assisted filling. Therefore, in order to reduce the filling time of HC-PBFs and thus the sensor response time, HC-PBF designs with wider core diameters or new measurement cell schemes using shorter fibre lengths are required.

## Figures and Tables

**Table 1.** Specifications of the HC-PBFs used in the experiments.

	HC1300	HC1600
Core diameter, *d* [*μ*m]	10	13.9
Fibre length, *L* [cm]	560	510
Pitch [*μ*m]	3	4.5
Rings in the cladding	7	7
Fibre diameter [*μ*m]	188	220
Bandgap [nm]	1150–1450	1375–1850^1^
Transmission loss [dB/km]	100	100

1 According to specifications.

**Table 2.** Comparative of the results of methane detection using HC1300 and HC1600 fibres.

	*ν*_2_ + 2*ν*_3_ band	2*ν*_3_ band
Fibre	HC1300	HC1600
Core diameter, *d* [*μ*m]	10.3	12
Fibre length, *L* [cm]	560	510
Transitions in the model	36	6
Linestrength, *S_i_* [cm*^−^*^2^atm*^−^*^1^]	0.004	0.033
Detection limit, *c_min_* [ppmv]	98	10
MDA [AU]	3.1 × 10*^−^*^3^	8.0 × 10*^−^*^4^
Filling time [min]	13	9.5

## References

[1] Culshaw B., Stewart G., Dong F., Tandy C., Moodie D. (1998). Fibre optic techniques for remote spectroscopic methane detection. Sens. Actuat. B.

[2] Chou J. (2000). Hazardous Gas Monitors: A Practical Guide to Selection, Peration and Applications.

[3] Lopez-Higuera J. (2002). Handbook of Optical Fibre Sensing Technology.

[4] Stewart G., Jin W., Culshaw B. (1997). Prospects for fibre-optic evanescent-field gas sensors using absorption in the near-infrared. Sens. Actuat. B.

[5] Ritari T., Tuominen J., Ludvigsen H., Petersen J., Sorensen T., Hansen T., Simonsen H. (2004). Gas sensing using air-guiding photonic bandgap fibers. Opt. Express.

[6] Kornaszewski L., Gayraud N., Stone J., Macpherson W., George A., Knight J., Hand D., Reid D. (2007). Mid-infrared methane detection in a photonic bandgap fiber using a broadband optical parametric oscillator. Opt. Express.

[7] Cubillas A., Lazaro J., Conde O., Petrovich M., Lopez-Higuera J. (2009). Multi-line fit model for the detection of methane at *ν*_2_ + 2*ν*_3_ band using hollow-core photonic bandgap fibres. Sensors.

[8] Cubillas A., Silva-Lopez M., Lazaro J., Conde O., Petrovich M., Lopez-Higuera J. (2007). Methane detection at 1670-nm band using a hollow-core photonic bandgap fiber and a multiline algorithm. Opt. Express.

[9] Austin E., van Brakel A., Petrovich M.N., Richardson D.J. (2009). Fibre optical sensor for C_2_H_2_ gas using gas-filled photonic bandgap fibre. Sens. Actuat B.

[10] Benabid F. (2006). Hollow-core photonic bandgap fibre: new light guidance for new science and technology. Phil. Trans. R. Soc. A.

[11] Petrovich M., van Brakel A., Poletti F., Musaka K., Austin E., Finazzi V., Petropoulos P., O’Driscoll E., Watson M., DelMonte T., Monro T., Dakin J., Richardson D. Microstructured fibres for sensing applications.

[12] Rothman L., Jacquemart D., Barbe A., Benner D., Birk M., Brown L., Carleer M., Chackerian C., Chance K., Coudert L., Dana V., Devi V., Flaud J., Gamache R., Goldman A., Hartmann J., Jucks K., Maki A., Mandin J., Massie S., Orphal J., Perrin A., Rinsland C., Smith M., Tennyson J., Tolchenov R., Toth R., Auwera J.V., Varanasi P., Wagner G. (2005). The HITRAN 2004 molecular spectroscopic database. J. Quant. Spectrosc. Radiat. Transf.

[13] Weldon V., O’Gorman J., Perez-Camacho J., Mcdonald D., Hegarty J., Corbett B. (1997). Methane sensing with a novel micromachined single-frequency Fabry-Perot lased diode emitting at 1331 nm. IEEE Phot. Tech. Lett.

[14] (2003). Physics of Atoms and Molecules.

[15] Quintela M., Quintela C., Lomer M., Madruga F., Conde O., Lopez-Higuera J. Comparison between a symmetric bidirectional-pumping and a unidirectional-pumping configurations in an erbium fiber ring laser.

[16] Thapa R., Knabe K., Corwin K., Washburn B. (2006). Arc fusion splicing of hollow-core photonic bandgap fibers for gas-filled fiber cells. Opt. Express.

[17] Gharavi M., Buckley S. (2005). Diode laser absorption spectroscopy measurement of linestrengths and pressure broadening coefficients of the methane 2*ν*_3_ band at elevated temperatures. J. Mol. Spec-trosc.

[18] Webber M., Kim S., Sanders S., Baer D., Hanson R., Ikeda Y. (2001). In situ combustion measurements of CO_2_ by use of a distributed-feedback diode-laser sensor near 2.0 *μ*m. Appl. Opt.

[19] Henningsen J., Hald J. (2008). Dynamics of gas flow in hollow core photonic bandgap fibers. App. Opt.

[20] Livesey R. (1998). Foundations of Vacuum Science and Technology: Flow of Gases through Tubes and Orifices.

[21] Trengove R., Wakeham W. (1987). The viscosity of carbon dioxide, methane and sulfur hexafluoride in the limit of zero density. J. Phys. Chem. Ref. Data.

